# Gukang Capsule Promotes Fracture Healing by Activating BMP/SMAD and Wnt/*β*-Catenin Signaling Pathways

**DOI:** 10.1155/2020/7184502

**Published:** 2020-09-29

**Authors:** Xue Ma, Jian Yang, Ting Liu, Jing Li, Yanyu Lan, Yonglin Wang, Aimin Wang, Ye Tian, Yongjun Li

**Affiliations:** ^1^State Key Laboratory of Functions and Applications of Medicinal Plants, Engineering Research Center for the Development and Applications of Ethnic Medicines and TCM (Ministry of Education), Guizhou Medical University, 4 Beijing Road, Guiyang 550004, China; ^2^Key Laboratory of Pharmaceutics of Guizhou Province, Guizhou Medical University, Guiyang 550004, China; ^3^School of Pharmacy, Guizhou Medical University, Guiyang 550004, China; ^4^Lab for Bone Metabolism, Key Lab for Space Biosciences and Biotechnology, School of Life Sciences, Northwestern Polytechnical University, 127 West Youyi Road, Xi'an 710072, China

## Abstract

**Background:**

Gukang capsule (GKC) is a traditional Chinese medicine formulation which has been used extensively in the clinical treatment of bone fractures. However, the mechanisms underlying its effects on fracture healing remain unclear.

**Methods:**

In this study we used a rabbit radius fracture model, and we measured the serum content of bone alkaline phosphatase (ALP), calcium, and phosphorus and examined pathology of the fracture site as indicators of the fracture healing effects of GKC. SaOS-2 human osteosarcoma cells were used to measure (i) ALP activity, (ii) ornithine transcarbamylase (OTC), calcium, and mineralization levels, (iii) the expression of osteogenic-related genes, that is, runt-related transcription factor 2 (RUNX2), bone morphogenetic protein 2 (BMP2), collagen I (COL-I), osteopontin (OPN), OTC, and osterix (Osx), and (iv) the expression of key proteins in the Wnt/*β*-catenin and BMP/SMAD signaling pathways to study the mechanisms by which GKC promotes fracture healing.

**Results:**

We found that GKC effectively promotes radius fracture healing in rabbits and enhances ALP activity, increases OTC and calcium levels, and stimulates the formation of mineralized nodules in SaOS-2 cells. Moreover, COL-I, OTC, Osx, BMP2, and OPN expression levels were higher in SaOS-2 cells treated with GKC than control cells. GKC upregulates glycogen synthase kinase 3*β* (GSK3*β*) phosphorylation and Smad1/5 and *β*-catenin protein levels, thereby activating Wnt/*β*-catenin and BMP/Smad signaling pathways. Inhibitors of the Wnt/*β*-catenin and BMP/Smad signaling pathways (DKK1 and Noggin, respectively) suppress the osteogenic effects of GKC.

**Conclusions:**

GKC promotes fracture healing by activating the Wnt/*β*-catenin and BMP/Smad signaling pathways and increasing osteoprotegerin (OPG) secretion by osteoblasts (OBs), which prevents receptor activator of nuclear factor kappa B ligand (RANKL) binding to RANK.

## 1. Background

Musculoskeletal fractures are very common, and almost one-third of humans suffer this disease during their lifetimes causing dramatic morbidity and motility [1, 2]. Selective estrogen receptor modulators (SERMs), parathyroid hormone (PTH_1-34_), bisphosphonates, and calcitonin are regularly used in the treatment of fractures [3]. However, long-term application of these drugs may cause many adverse reactions. For example, long-term application of PTH_1-34_ may increase the risk of osteosarcoma [4]. Multicomponent, multitarget, and low-toxicity traditional Chinese medicines (TCMs) have been used extensively in the clinical treatment of fractures in China and have been proved to possess many advantages [5, 6].

GKC is a traditional TCM formulation consisting of *Fructus Psoraleae*, *Dipsacus asper* Wall*., Panax notoginsen*g Wall., *Musa basjoo* Siebold, and *Oxalis corniculata* Linn. It strengthens tendons and bones, nourishes the liver and kidney, and stimulates meridians to achieve pain relief according to TCM theory. GKC is commonly used as a supplementary treatment for fracture in clinical practice in China and shows good therapeutic effects [7]. However, the mechanisms underlying its effects in promoting fracture healing have not been clarified, affecting the further application of GKC.

Fracture healing is a dynamic biological process that involves restoration of the original tissue structure and bone function via complex physiological processes and the interaction of various types of bone cells, cytokines, and genes [8]. During fracture healing process, osteoblasts (OBs) are coupled with osteoclast and remodel the callus tissues to the bone's original cortical structure [8]. OBs are the key cells involved in new bone formation at fracture gap. OBs are mainly responsible for the synthesis and secretion of bone matrix, which further matures and mineralizes to form new bone tissue. In the process of osteogenesis, OBs go through four stages, that is, proliferation, extracellular matrix maturation, extracellular matrix mineralization, and apoptosis. Some hormones and cytokines affect osteogenesis by regulating the above four stages of OBs [9, 10].

A previous study has shown that the canonical Wnt signaling pathway is closely related to the proliferation and differentiation of OBs [11]. When the Wnt protein is present extracellularly, it binds to the corresponding receptor on the cell membrane, and the signal is transmitted by the key protein GSK3*β*. The phosphorylation of GSK3*β* in Ser 9 results in an increase in the amount of *β*-catenin in the cytoplasm; *β*-catenin ultimately enters the nucleus, where it interacts with T-cell factor/lymphoid enhancer factor (TCF/LEF), activates the downregulated RUNX2 gene, and promotes the Osx gene to initiate transcription of a series of target gene, thereby promoting the proliferation and differentiation of OBs [12, 13]. In addition, BMPs play multiple roles in body development, especially in the bone development and bone tissue formation; they have a strong ability to induce OB differentiation and osteogenesis [14]. BMPs mainly exert their biological functions through the canonical BMP signaling pathway [15]. BMPs bind to type I and type II BMP receptors to activate the type I receptor. The activated type I receptor further phosphorylates the cytoplasmic signaling molecules Smad1/5/8. These phosphorylated Smad1/5/8 bind to Smad4 to form a complex and enter the nucleus, where they regulate the transcription of related target genes [16, 17].

Our previous study showed that GKC promoted the osteogenic differentiation and mineralization of SaOS-2 human osteosarcoma cells. However, the mechanisms underlying its effects on bone differentiation and mineralization have not been clarified. In this study, we used a rabbit radius fracture model to confirm that GKC promoted fracture healing *in vivo*, and we used a SaOS-2 cell line to study the mechanisms by which the Wnt/*β*-catenin and BMP/Smad signaling pathways affect osteogenesis upon GKC treatment. In addition, given that many TCMs have multiple targets, we also examined the effects of GKC on the osteoprotegerin (OPG)/receptor activator of nuclear factor kappa B ligand (RANKL) system.

## 2. Materials and Methods

### 2.1. Reagent

SaOS-2 cell was kept in the laboratory. GKC (20180521) was obtained from Guizhou Wei Kang Zi Fan Pharmaceutical Co. Ltd. (Guiyang, China). Jiegu-Qili tablet was obtained from Hunan Jin Sha Pharmaceutical Co. Ltd. (Changsha, China). Pentobarbital Sodium (6900183) was purchased from Beijing Solarbio Science & Technology Co. Ltd. Penicillin G Sodium (170907) was from Lukang Pharmaceutical Co. Ltd. (Jining, China). ALP (A059-2), Pi (C006-3), and Ca (C004-2) were supplied by Nanjing Jiancheng Biotechnology Co. Ltd. (Nanjing, China). Primers of ALP, COL-I, OTC, Osterix, RUNX2, BMP2, OPN, OPG, RANKL, and GAPDH were obtained from Shanghai Generay Biotech Co. Ltd. (Shanghai, China). Antibodies against RUNX-2 (ab23981), OPG (ab73400), BMP2 (ab14933), RANKL (ab9957), *β*-catenin (ab6302), Smad4 (ab40759), GAPDH (ab8245), and GSK3*β* (ab93926) were from Abcam (Cambridge, England). DKK1 (PHC9214), Noggin (PHC1506), antibodies against Smad1/5 (PA5-80036), and p-Smad1/5 (MA5-15124) were obtained from Thermo fisher (American). p-GSK3*β* (Ser9, 9336S) antibody was got from Cell Signaling Technology (American). All other reagents used in the present study were of analytical grade.

### 2.2. Animals

Male Healthy rabbits (2.0–2.5 kg) were purchased from Chongqing Teng Xin Biotechnology Co. Ltd. (Chongqing, China). The license number for animal manufacturers SCXK(Yu)2012-0003. Rats were maintained at a controlled temperature (22 ± 2°C) and humidity (50 ± 5%) under a 12 h light/dark cycle and given free access to water and food. All experiments were carried out under the approval of the Animal Ethical Committee of Guizhou Medical University (allowance number: 1702085) and conformed with the guidelines of the National Institutes of Health for the Care and Use of Animals.

### 2.3. Fracture Model Preparation and Drug Administration

**Figure 1:**
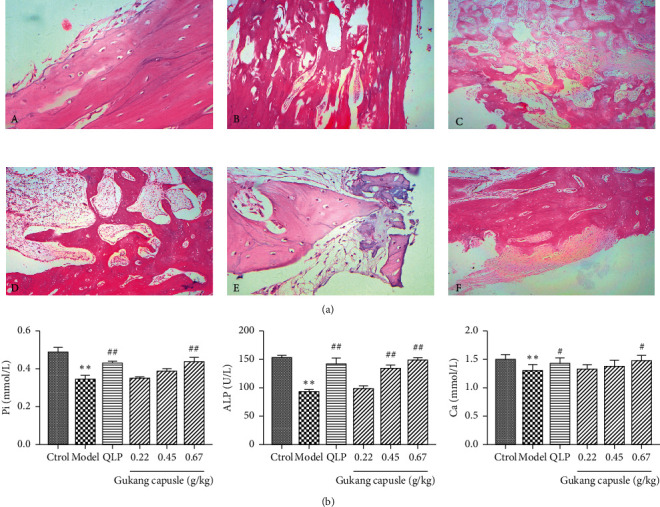
GKC promotes fracture healing. (a) Representative images of the pathological sections of the fracture gaps (hematoxylin and eosin staining, ×400 magnification): (A) control group; (B) model group; (C) Jiegu-Qili tablet group; (D) low-dose GKC group; (E) moderate-dose GKC group; and (F) high-dose GKC group. (b) Measurement of serum ALP, calcium, and phosphorus levels in rabbits. ^*∗∗*^*P* < 0.01, relative to the control group; ^#^*P* < 0.05 and ^##^*P* < 0.01, relative to the GKC groups.

Rabbits were anesthetized by intravenous injection of 3% sodium pentobarbital (30 mg/kg) through the ear vein. Each rabbit was then fixed on the operation bench, and the hair of the left forelimb was shaven off. The skin was disinfected with 75% ethanol. A sterile scalpel was used to cut open the skin and separate the surrounding muscle, soft tissues, and blood vessels to fully expose the radius. A small electric saw was used to induce a fracture in the bone, resulting in a complete defect of 3 mm in the middle of the left upper limb (Figure S1). The ulna was kept intact. Penicillin G sodium (40,000 units) was instilled into the incision site to avoid infection. Bleeding was stopped, the wound was washed with normal saline, and the skin was sutured layer by layer, as shown in Figure 1(a). Sterile gauze was used to wrap the wound without plaster splint fixation. Each animal was intramuscularly injected with 40,000 units of penicillin once a day for the following three days.

### 2.4. Animal Grouping and Drug Administration

42 male rabbits were randomly divided into six groups (*n* = 7 per group), that is, the control group, model group, Jiegu-Qili tablet group (0.14 g/kg), low-dose GKC group (0.22 g/kg), moderate-dose GKC group (0.45 g/kg), and high-dose GKC group (0.67 g/kg). Treatments were administered by oral gavage once per day, with rabbits of the control and the model group being administered equal amounts of distilled water.

### 2.5. Measurement of Serum ALP, Calcium, and Phosphorus in Rabbits

Three milliliters of blood were taken from the heart of each rabbit 30 days after establishment of the model. The blood samples were stored at 4°C for approximately 2 h, until blood clotting. The clotted blood samples were centrifuged at 3,000 rpm for 10 min, and the serum was carefully collected and stored at −80°C for later use. The measurement of serum ALP, calcium, and phosphorus was performed in strict accordance with the instructions of the manufacturers of the corresponding assay kits.

### 2.6. Pathological Examinations of Rabbit Fracture

After establishing the model for 30 days, all rabbits were euthanized. The left radius (with fracture) was completely dissected and sawn 1 cm away from both ends of the fracture site, the surrounding skeletal muscles and soft tissues were removed, and the bone was fixed in 10% formaldehyde solution. The fixed bone tissues were placed in acid mixed decalcifying solution (800 ml distilled water + 100 ml hydrochloric acid + 100 ml formic acid) and were left to decalcify for a week. Decalcification was considered complete if a pin could easily penetrate into the bone tissue. The decalcified bone tissues were subjected to conventional paraffin embedding, sectioning, hematoxylin and eosin (HE) staining, and light microscopy.

### 2.7. Cell Culture and Treatment

SaOS-2 human osteosarcoma cells were cultured in McCoy's 5a medium containing 10% fetal bovine serum and 1% penicillin/streptomycin in an incubator with 5% CO_2_ at 37°C until 80% confluency was reached. The cells were then trypsinized and subcultured. Well-grown cells of passage 5–10 in the logarithmic growth phase were used for subsequent experiments.

The cytotoxicity of different concentrations of GKC on SaOS-2 cells were measured by MTT assay. Concentrations of 10, 100, 200, and 1,000 mg/l for 1, 3, and 5 days were not cytotoxic. SaOS-2 cells in the logarithmic growth phase were selected, and cell suspension of 2 × 10^5^ cells/ml was inoculated in a 24-well plate (500 *μ*L/well); after 24 h, the medium was refreshed. To monitor the effect of GKC to the differentiation of SaOS-2 cells, the culturing medium was changed to osteogenic osteoinduction medium which contained 10 mM *β*-glycerophosphate, 10 mM dexamethasone, 50 *μ*g/mL ascorbic acid, and GKC of different concentrations. SaOS-2 cells of the low-dose, moderate-dose, and high-dose GKC groups were treated with medium containing 10, 100, and 200 mg/l GKC, respectively. SaOS-2 cells of the GKC plus DKK1 group were treated with medium containing DKK1 at a final concentration of 0.5 *μ*g/ml for 5 days. Then the medium was replaced with medium containing 200 mg/l GKC. SaOS-2 cells of the GKC plus Noggin group were first treated with medium containing 1.5 *μ*g/ml Noggin for 5 days. Then the medium was replaced with medium containing 200 mg/l GKC. The control group were incubated with McCoy's 5a medium. The vitamin D_3_ group were incubated with medium containing 30 mg/l vitamin D_3_.

### 2.8. Measurement of ALP Activity

After 48 h of treatment of SaOS-2 cells, the culture medium was discarded, the cells were washed twice with phosphate-buffered saline (PBS), 200 *μ*l 1% Triton X-100 was added, and the cells were kept on ice for cell lysis for 10 min. The ALP activity on SaOS-2 cells was measured by disodium phenyl phosphate colorimetry. ALP activity and protein quantity were measured by ALP kit and bicinchoninic acid (BCA) assay, respectively.

### 2.9. ALP Staining

Seven days after treatment of SaOS-2 cells, the culture medium was discarded, the cells were washed twice with PBS, and the cells were fixed with 95% ethanol for 15 min. The cells were washed three times with PBS, an appropriate amount of 5-bromo-4-chloro-3′-indolyphosphate p-toluidine salt (BCIP)/nitro-blue tetrazolium chloride (NBT) staining solution was added, and samples were incubated at room temperature for 30 min in the dark. After removing the staining solution and washing twice with distilled water, the stained cells were observed by light microscopy.

### 2.10. Mineralized Nodule Staining

Fourteen days after treatment of SaOS-2 cells, the medium was discarded, the cells were washed twice with PBS, and the cells were fixed with 95% ethanol for 10 min. The cells were washed three times with distilled water, an appropriate amount of 0.1% Alizarin Red (pH 8.3)-Tris-HCl staining solution was added, and the cells were incubated for 30 min. Excess dye was removed by washing with distilled water, and the cells were air-dried and observed and photographed under a light microscope.

### 2.11. Real-Time Quantitative PCR (qRT-PCR)

Twenty-four hours after treatment of SaOS-2 cells, total RNA was extracted using TRIzol reagent, followed by reverse transcription using PrimeScript™ RT reagent Kit (TaKaRa). The target gene expression was quantified by SYBR Green II-based qRT-PCR, and the 2^−ΔΔCt^ method was used to calculate the fold change compared to the control group.  Table 1 shows the primer sequences used in the qRT-PCR, and Table 2 shows the reaction procedures of the qRT-PCR. After the reaction was completed, the amplification and dissolution curves were drawn and confirmed. The number of cycles (Ct) of the sample to be tested was analyzed and calculated, and the relative mRNA expression of each target gene was calculated using the 2^−ΔΔCt^ method.

### 2.12. Western Blot Analysis

24 hours after treatment of SaOS-2 cells, the cells were lysed with RIPA buffer, followed by protein quantification by BCA assay. 30 *μ*g of each sample of SaOS-2 cells was loaded per lane for separation by SDS-PAGE. Protein was transferred to PVDF membranes, and membranes were blocked in blocking buffer (5% bovine serum albumin) at 4°C overnight. The primary antibodies were diluted in blocking buffer according to the corresponding dilution factors. Membranes were incubated with primary antibody on a shaker at 4°C for 2 h, washed with Tris-buffered saline with 0.1% Tween 20 (TBST) five times (5 min each), and incubated with HRP-conjugated secondary antibody (diluted in TBST buffer) on a shaker at room temperature for 2 h. After washing with TBST five times (5 min each), the membranes were photographed using a gel imager. The scanned images were analyzed, and the gray value ratio of target protein to GAPDH in each lane was analyzed separately to calculate the relative protein expression levels.

### 2.13. Statistical Analysis

Statistical analyses were carried out using SPSS13.0 (IBM SPSS Inc., Chicago, IL). Data were compared by one-way ANOVA. Results are presented as mean ± standard deviation, and *P* < 0.05 was considered to indicate a statistically significant difference.

## 3. Results

### 3.1. GKC Promotes Fracture Healing in Rabbits

The pathological sections of the fracture gaps (Figure 1(a)) showed that the bone tissue structure of the rabbits in the control group was normal. In the model group, the bone trabecula at the fracture end was slender and sparse and was connected with the fibrotic tissue. For drug treatment group, the calluses were composed of fibroblasts, cartilage and newly formed trabecular bone, and more regular arrangement of collagen fibers and more bone trabeculae were observed. GKC groups exhibited progressive mineralized callus formation with the increment of doses compared to model group and Jiegu-Qili tablet group. Thicker and more mature bone trabeculae were discovered compared to Jiegu-Qili tablet group. Especially for the highest GKC group, significantly more mineralized and mature callus were displayed compared to other groups.

As shown in Figure 1(b), the blood phosphorus content of GKC-treated rabbits increased with doses, and the blood phosphorus content of the high-dose (0.67 g/kg) GKC group was significantly higher than that of the model group (*P* < 0.01). Comparison of serum bone ALP levels between the GKC groups and the model group has been made. It showed no significant difference between the low-dose (0.22 g/kg) GKC group and the model group; however, the serum ALP levels of the moderate-dose (0.45 g/kg) and high-dose Gukang groups were significantly higher than the model group (*P* < 0.01). In addition, the calcium content of the GKC groups was higher than that of the model group, with a significant difference between the high-dose GKC group and the model group (*P* < 0.05).

### 3.2. GKC Promotes Osteogenic Differentiation of SaOS-2 Cells

**Figure 2:**
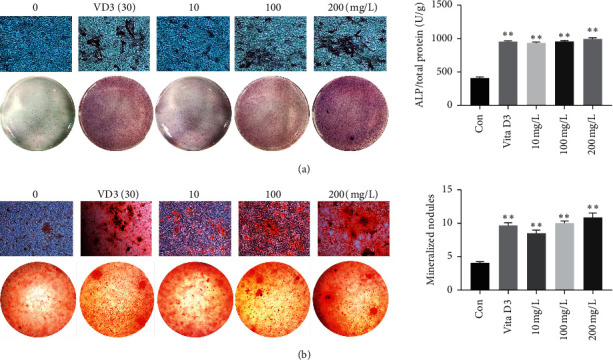
Effects of GKC on the osteogenic differentiation and mineralization of SaOS-2 cells. (a) ALP staining and activity detection (*n* = 3). ^*∗∗*^*P* < 0.01, relative to the control group. (b) Mineralized nodule staining and ALP activity assay (*n* = 3). ^*∗∗*^*P* < 0.01, relative to the control group.

To further explore the effects of GKC in promoting osteogenesis and osteogenic differentiation in SaOS-2 cells, we performed staining and quantitative analysis of ALP and mineralized nodules. Our results showed that as the concentrations of GKC increased, the ALP staining intensity of the GKC groups also increased. Analogous phenomenon was observed in MC3T3-E1 cells as well (Figure S2). Compared to the control group, ALP activity was significantly increased in the GKC groups in a dose-dependent manner (Figure 2(a)). Similar observations were made for staining and quantitative analysis of mineralized nodules in SaOS-2 cells (Figure 2(b)).

### 3.3. Effects of GKC on the Expression of Genes Related to Osteogenic Functions and the OPG/RANKL System in SaOS-2 Cells

**Figure 3:**
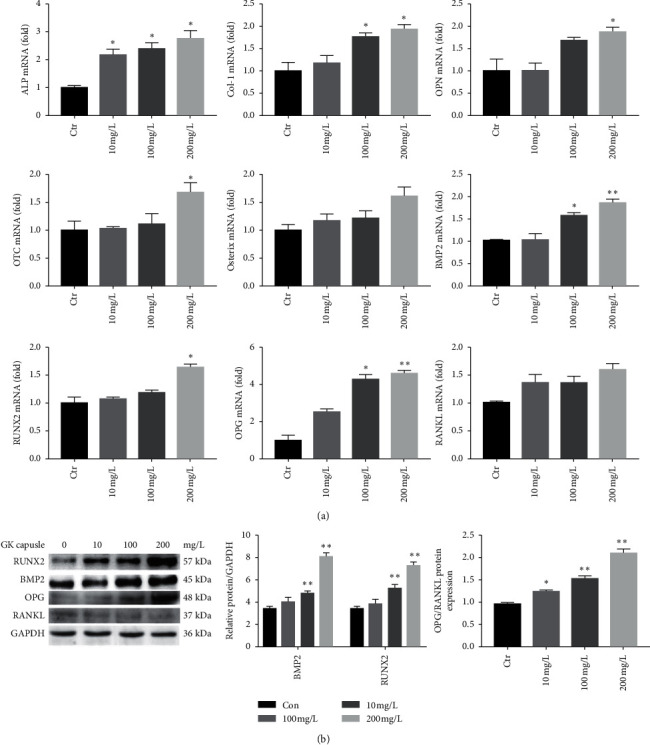
Effects of GKC on the osteogenic differentiation of SaOS-2 cells. (a) RT-qPCR was used to quantify the mRNA expression of osteogenic marker genes ALP, COL-I, OTC, OPN, Osx, RUNX2, BMP2, OPG, and RANKL after 24 h of treatment with different concentrations of GKC. The mRNA expression levels were normalized to the mRNA expression of GAPDH (*n* = 3). ^*∗*^*P* < 0.05 and ^*∗∗*^*P* < 0.01, relative to the control group. (b) Effects of 24 h of treatment with different concentrations of GKC on the protein expression of osteogenic markers, as analyzed by western blot analysis (*n* = 3). ^*∗*^*P* < 0.05 and ^*∗∗*^*P* < 0.01, relative to the control group.

Figure 3(a) showed the effect of GKC on the expression of genes related to osteogenic functions. The mRNA expression levels of *ALP*, *COL-I*, *OPN*, *OTC*, *RUNX2*, *BMP2*, and *OPG* were generally increased after 24 h of GKC treatment (*P* < 0.05), and ALP mRNA changed most (*P* < 0.05). GKC also boosted the *Osx* mRNA expression, but no significant difference was found compared to the control group (*P* > 0.05). GKC had no significant effect on *RANKL* mRNA expression *in vitro*.

Western blot analysis (Figure 3(b)) showed that BMP2 and RNX2 protein levels gradually increased as the concentration of GKC increased. This result is consistent with the changes in *BMP2* and *RUNX2* mRNA expression in GKC-treated SaOS-2 cells. GKC (10 mg/l, 100 mg/l, and 200 mg/l) significantly increased the OPG/RANKL protein ratio, and 200 mg/l had the greatest effect on the OPG/RANKL ratio.

### 3.4. Wnt/*β*-Catenin Signaling Pathway Is Involved in the GKC-Induced Osteogenic Differentiation of SaOS-2 Cells

**Figure 4:**
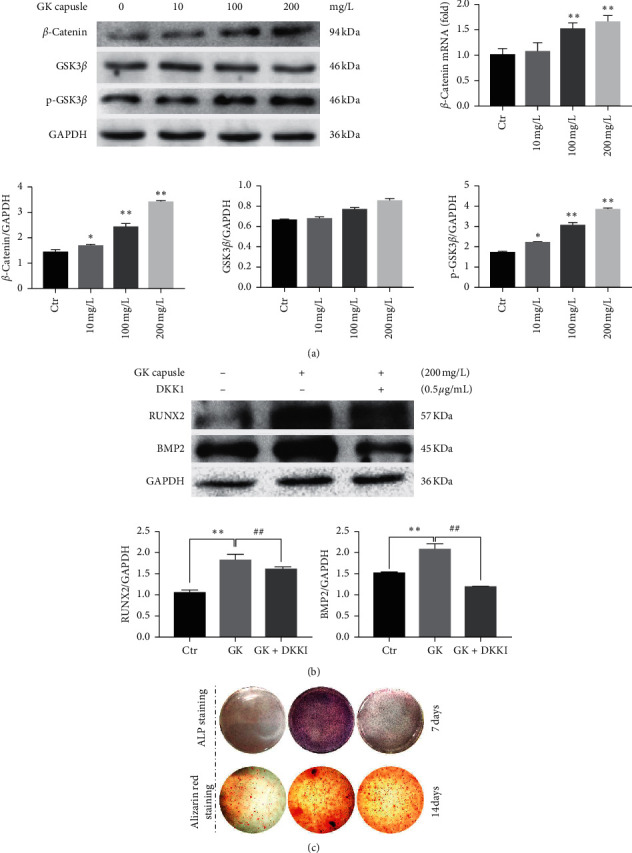
Effects of GKC on Wnt/*β*-catenin signaling during the osteogenic differentiation of SaOS-2 cells. (a) RT-qPCR was used to measure mRNA expression levels of the Wnt/*β*-catenin signaling pathway-related key gene *β*-catenin, and western blot analysis was used to measure the protein levels of *β*-catenin, GSK3*β*, and phosphorylated GSK3*β* after 24 h of GKC treatment at different concentrations (*n* = 3). (b) Representative western blots showing BMP2 and RUNX2 protein expression of SaOS-2 cells, with or without DKK1 pretreatment (5 h 0.5 *μ*g/ml), treated with or without 200 mg/l GKC for 24 h (*n* = 3). (c) ALP staining (7 days) and Alizarin Red staining (14 days) of SaOS-2 cells after DKK1 treatment (*n* = 3). ^*∗*^*P* < 0.05 and ^*∗∗*^*P* < 0.01, relative to the control group. ^#^*P* < 0.01, relative to the GKC treatment groups.

Studies of the Wnt/*β*-catenin signaling pathway showed that after 24 h of GKC treatment, *β*-catenin mRNA, and protein expression levels were increased in a dose-dependent manner, and the phosphorylation of GSK3*β* protein was effectively upregulated (Figure 4(a)). In addition, Dikkopf-1 (DKK1), an inhibitor of the Wnt/*β*-catenin signaling pathway, significantly suppressed the GKC-induced RUNX2 and BMP2 protein expression (Figure 4(b)) as well as ALP activity and mineralization in SaOS-2 cells (Figure 4(c)).

### 3.5. BMP/Smad Signaling Pathway Is Also Involved in the GKC-Induced Osteogenic Differentiation of SaOS-2 Cells

**Figure 5:**
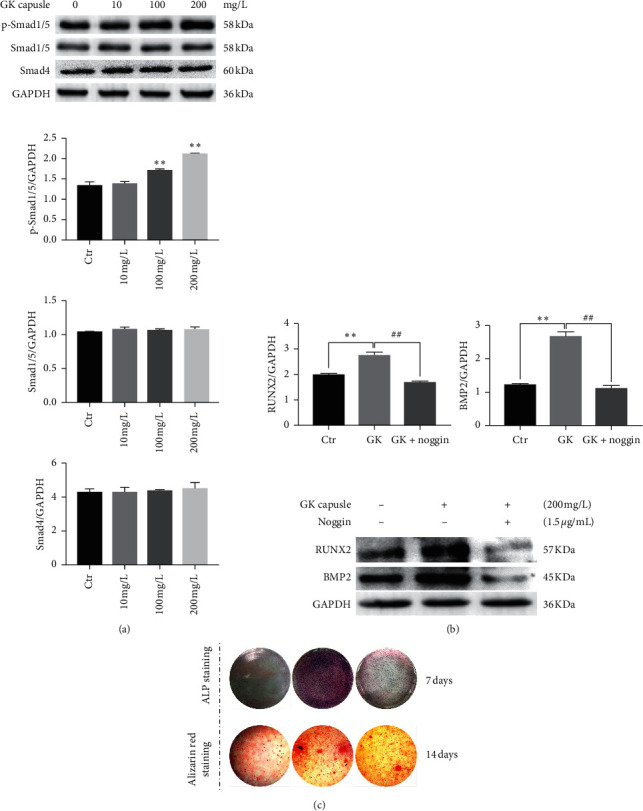
GKC activates the BMP/Smad signaling pathway, promoting osteogenic differentiation of SaOS-2 cells. (a) Western blot showing the levels of phosphorylated Smad1/5, Smad1/5, and Smad4 after 24 h of treatment with GKC at different concentrations (*n* = 3). (b) BMP2 and RUNX2 protein expression were downregulated in SaOS-2 cells treated with 1.5 *μ*g/ml Noggin for 5 h (*n* = 3). (c) ALP staining (7 days) and Alizarin red staining (14 days) of SaOS-2 cells showed that Noggin treatment reverses the effects of GKC on ALP activity and the formation of mineralized nodules (*n* = 3). ^*∗*^*P* < 0.05, relative to the control group. ^#^*P* < 0.01, relative to the GKC treatment groups.

Compared to the control group, 24 h of GKC treatment increased the phosphorylation levels of Smad1/5 in SaOS-2 cells in a dose-dependent manner. Significant differences were detected at doses of 100 mg/l and 200 mg/l (*P* < 0.01). However, GKC had no significant impact on total Smad1/5 and Smad4 protein expression (*P* > 0.05, Figure 5(a)). The BMP pathway inhibitor Noggin reduced the RUNX2 and BMP2 protein levels (Figure 5(b)) and reversed the effects of GKC in promoting ALP activity and the formation of mineralized nodules (Figure 5(c)).

## 4. Discussion

TCMs have been applied widely for prevention and treatment of many bone diseases including fractures for [18]. They are more suitable for long-term use and are alternative medicines owing to their safe and cost-effective features [6, 19]. GKC, which is composed of the extracts of five herbal medicine, is in bud of orthopedic treatment. Our study is the first to investigate the effects of GKC on fracture healing in a rabbit model and related mechanisms in SaOS-2 cells. We found the promotion effect of GKC on fracture healing was concerned about Wnt/*β*-catenin and BMP/Smad signaling pathways thus enhanced OBs function. Jiegu-Qili tablet has been proved to promote fracture healing in rabbit [20] and rat [21] models and is widely used in treatment for fracture in clinical practice [22, 23]. Therefore, we chose it as positive control to evaluate the efficacy of GKC on fracture impairment. According to our results, GKC had higher activity for accelerating the cortical bone remodeling and the union of fracture, especially at high dose. These findings suggest that GKC is very promising in fracture treatment clinically.

OBs induce and regulate the mineralization of extracellular matrix through their own proliferation and differentiation, thereby regulating the process of bone remodeling during fracture healing. Pending the continuous proliferation of OBs and the formation of multiple layers of cells, COL-I and several noncollagen proteins are synthesized, secreted, and subsequently mineralized to form bone nodules that eventually become intact bone [24]. GKC significantly upregulates the mRNA and protein expression of RUNX2 and BMP2 in OBs. It promotes mRNA expression of several osteogenesis marker genes, that is, COL-I, OPN, OTC, and Osx, in OBs, suggesting that GKC promotes osteogenesis in SaOS-2 cells.

ALP is abundant in the cytoplasm of OBs. It is used as an OB marker and as an indicator of the degree of OB differentiation. Serum ALP content represents an increase in OB activity and is an indication for the activity of bone remodeling [25]. In the process of fracture healing, serum calcium and phosphorus directly affect bone calcification, and increased serum calcium and phosphorus levels contribute to bone salt deposition in a healing fracture [26]. We have shown that GKC significantly upregulated the serum ALP levels in a rabbit fracture model and impacted serum calcium and phosphorus levels, suggesting that GKC promotes fracture healing by promoting osteogenic activity of OBs.

Wnt/*β*-catenin signaling plays an important role in the regulation of osteogenesis and bone resorption in the body; *β*-catenin is the core and key regulator of the canonical Wnt/*β*-catenin signaling pathway. Its concentration in the cytoplasm is determined by the functional state of the dynamic degradation complex. When the extracellular Wnt proteins of OBs bind to the frizzled protein and coreceptor LRP5/6 on the cell membrane, dimers are formed under the action of the relevant membrane and cytoplasmic proteins, causing the inactivation of GSK-3*β* (phosphorylation in Ser 9 is a hallmark of GSK3*β* inhibition), thereby inhibiting the degradation of *β*-catenin. Hence, *β*-catenin gradually accumulates in the cytoplasm. Once *β*-catenin enters the nucleus, it interacts with the nuclear transcription factor TCF/LEF, activating the expression of the downstream genes RUNX2 and Osx, thereby initiating transcription of a series of related target genes, and promoting OB proliferation and differentiation [27]. Blockade of Wnt signaling causes GSK3*β* binding to the free *β*-catenin in the cytoplasm, resulting in the phosphorylation and degradation of *β*-catenin. Therefore, GSK3*β* is an important negative regulator in the Wnt/*β*-catenin signaling pathway [28]. DKK1 is an inhibitor of the canonical Wnt/*β*-catenin signaling pathway. It inhibits Wnt signaling by competing with the ligand Wnt for receptor LRP5/6 and affects the expression of downstream genes that play a role in osteogenic differentiation.

In addition to Wnt/*β*-catenin signaling, several signaling pathways are involved in the expression or activation of key transcription factors, such as RUNX2, which regulate OB differentiation and osteogenesis. Among them, the most important key transcription factor is the BMP/Smad signaling pathway [29, 30]. BMP2 bind to specific receptors BMP-I and BMPR-II on the cell membrane to phosphorylate BMPR-I and then bind to Smad1/5/8, which specifically interact with BMPs, leading to phosphorylation of Smad1/5/8. Phosphorylated Smad1/5/8 bind to Smad4 in the cytoplasm and then enter the nucleus to continue to regulate the expression of downstream OB-specific transcription factors, such as RUNX2 and Osx, promote osteogenic differentiation, and accelerate osteogenesis [31].

In this study, we showed that the expression of *β*-catenin, a key factor in the Wnt/*β*-catenin signaling pathway, dose-dependently increased in SaOS-2 cells treated with GKC. GKC also upregulated the phosphorylation level of GSK3*β* protein in SaOS-2 cells. In addition, GKC upregulated the phosphorylation level of Smad1/5 in the BMP/Smad signaling pathway. The Wnt/*β*-catenin and BMP/Smad signaling pathway inhibitors DKK1 and Noggin reversed the inhibitory effect of GKC on ALP activity and the formation of mineralized nodules, indicating that GKC promoted OB differentiation through BMP/Smad and Wnt/*β*-catenin signaling. Previous studies had shown that BMP/Smad and Wnt/*β*-catenin signaling cooperated and promoted each other's function during OB differentiation and maturation [32, 33]; *β*-catenin plays a role in the regulation of BMP2 protein expression, and activation of the Wnt/*β*-catenin signaling pathway effectively promoted BMP2 gene expression. Activation of the BMP/Smad signaling pathway increases the expression of membrane receptors and ligand proteins in the Wnt/*β*-catenin signaling pathway, inhibiting the degradation of cytoplasmic *β*-catenin. As a result, the Wnt/*β*-catenin signaling pathway exhibits a stronger ability to regulate osteogenic differentiation [34]. In this study, the mRNA and protein expression levels of *β*-catenin, BMP2, and RUNX2 were significantly upregulated in the SaOS-2 cells treated with GKC. Therefore, the osteogenic effects of GKC in SaOS-2 cells are most likely jointly mediated by the Wnt/*β*-catenin and BMP/Smad signaling pathways.

The OPG/RANK/RANKL system plays an important role in maintaining the dynamic balance of bone tissue in the body. OPG is a member of the tumor necrosis factor receptor superfamily. It is the only factor that is known to directly downregulate the function of osteoclasts, and it inhibits their activation and differentiation. Therefore, OPG is also known as osteoclastogenesis inhibitory factor. OPG content is a direct measure of bone resorption activity. RANKL is mainly secreted by OBs and bone matrix. It is a member of the tumor necrosis factor superfamily. RANKL binds to RANK on the surface of osteoclasts to promote the differentiation and maturation of osteoclasts. In addition, RANKL inhibits the apoptosis of osteoclasts, thereby promoting osteoclastic bone resorption [35]. OPG competes for the binding site of RANK on RANKL and hence prevents RANK binding, thereby blocking osteoclast-induced differentiation, survival, and fusion of osteoclast precursors, inducing osteoclast apoptosis, and inhibiting the activation of mature osteoclasts [36]. Thus, the OPG/RANKL ratio is a decisive factor in the regulation and evaluation of bone resorption and bone remodeling. GKC promoted OPG expression and upregulated the OPG/RANKL ratio, suggesting that GKC also had a preventive effect on bone resorption.

## 5. Conclusions

In summary, we used a rabbit radius fracture model to confirm that GKC promoted fracture healing *in vivo.* Besides, a SaOS-2 cell line was used to study the mechanisms, and we found that GKC promoted fracture healing through enhancing osteoblast differentiation and triggering the Wnt/*β*-catenin and BMP/Smad signaling pathways. In addition, given that many TCMs have multiple targets, we also examined the effects of GKC on the OPG/RANKL system, and the results showed that GKC increased OPG secretion by OBs, which prevents RANKL binding to RANK.

## Figures and Tables

**Table 1:** qRT-PCR primers sequences for related genes.

Genes	Forward primer sequence	Reverse primer sequence	Accession number
ALP	ACCTCGTTGACACCTGGAAG	CCACCATCTCGGAGAGTGAC	NM_001632.4
COL-I	GACTGCCAAAGAAGCCTTGCC	TTCCTGACTCTCCTCCGAACCC	NM_000088.3
OTC	GACTGTGACGAGTTGGCTGA	CTGGAGAGGAGCAGAACTGG	NM_199173.5
Osterix	CGGGACTCAACAACTCT	CCATAGGGGTGTGTCAT	NM_001173467.2
RUNX2	TTACTTACACCCCGCCAGTC	TATGGAGTGCTGCTGGTCTG	NM_001015051.3
BMP2	ACTCGAAATTCCCCGTGACC	CCACTTCCACCACGAATCCA	NM_001200.3
OPN	CTCCATTGACTCGAACGACTC	CAGGTCTGCGAAACTTCTTAGAT	NM_000582.2
OPG	GAACCCCAGAGCGAAATACA	CGCTGTTTTCACAGAGGTCA	NM_002546.3
RANKL	AGAGCGCAGATGGATCCTAA	TTCCTTTTGCACAGCTCCTT	NM_003701.3
*β*-catenin	TGGTGCCCAGGGAGAACCCC	CCCACCCCTCGAGCCCTCTC	NM_001098209.1
GAPDH	GCACCGTCAAGGCTGAGAAC	ATGGTGGTGAAGACGCCAGT	NM_001256799.2

**Table 2:** PCR amplification program.

	Procedure	Annealing temp (°C)	Time	Cycle number
Stage 1	Initial denaturation	95	30 s	1
Stage 2	PCR reaction	95	3 s	40
		60	30 s	
	Plate read			
Stage 3	Melt curve stage			

## Data Availability

The data used to support the findings of this study are available from the corresponding author upon request.

## Supplementary Materials

Supplementary Materials Figure S1: establishment of the rabbit radius fracture model: (a) skin disinfection; (b) exposure of the radius; (c) inducing fracture; and (d) skin suturing. Figure S2: ALP staining of MC3T3-E1 cells treated with GKC of different concentrations.
