# Supplementary material for: Gukang Capsule Promotes Fracture Healing by Activating BMP/SMAD and Wnt/β-Catenin Signaling Pathways
Source: Evid Based Complement Alternat Med. 2020 Sep 29;2020:7184502. doi: 10.1155/2020/7184502 (PMC7545469; doi:10.1155/2020/7184502)
Supplement: Supplementary Materials — Figure S1: establishment of the rabbit radius fracture model: (a) skin disinfection; (b) exposure of the radius; (c) inducing fracture; and (d) skin suturing. Figure S2: ALP staining of MC3T3-E1 cells treated with GKC of different concentrations. [file 7184502.f1.docx]

**Gukang Capsule Promotes Fracture Healing by Activating** **BMP/SMAD and Wnt/β-catenin Signaling Pathways**

**Xue Ma^1^**^†^**, Jian Yang^1^**^†^**, Ting Liu^2^, Jing Li^2,4^, Yanyu Lan^1^, Yonglin Wang^2^, Aimin Wang^1^, Ye Tian^3*^, Yongjun Li^1*^**

***** Correspondence: [tianye@nwpu.edu.cn;](mailto:tianye@nwpu.edu.cn;) [liyongjun026@gmc.edu.cn](mailto:liyongjun026@gmc.edu.cn)

† Xue Ma and Jian Yang contributed equally to this work

^1^ State Key Laboratory of Functions and Applications of Medicinal Plants, Engineering Research Center for the Development and Applications of Ethnic Medicines and TCM (Ministry of Education), Guizhou Medical University, 4 Beijing Road, Guiyang 550004, China

^2^Key Laboratory of Pharmaceutics of Guizhou Province, Guizhou Medical University, Guiyang 550004;

^3^ Lab for Bone Metabolism, Key Lab for Space Biosciences and Biotechnology, School of Life Sciences, Northwestern Polytechnical University, 127 West Youyi Road, Xi’an, 710072, China

^4^ School of Pharmacy, Guizhou Medical University, Guiyang 550004, P.R. China

**Supplementary Files**

**
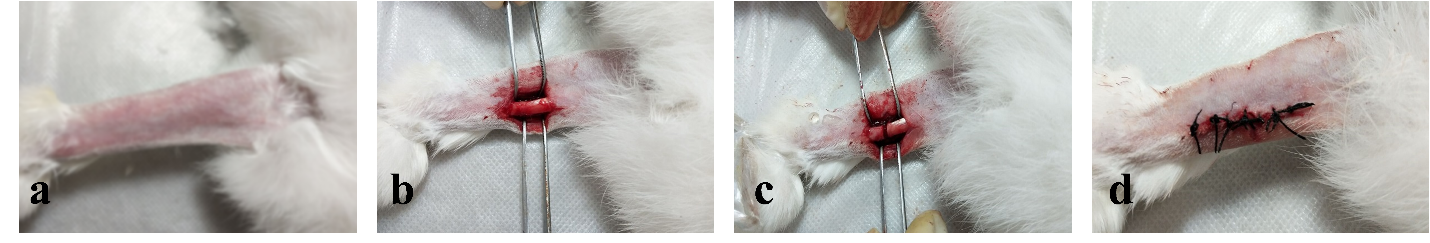
**

Figure S1. Establishment of the rabbit radius fracture model: (a) skin disinfection; (b) exposure of the radius; (c) inducing fracture; and (d) skin suturing.


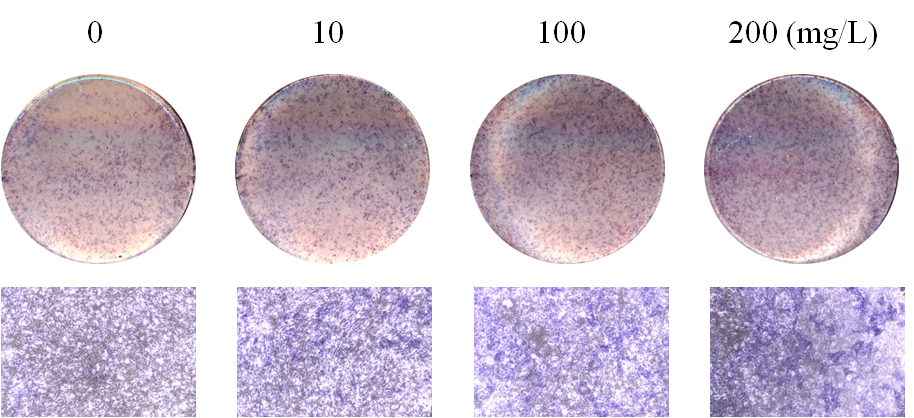


Figure S2. ALP staining of MC3T3-E1 cells treated with GKC of different concentrations.
